# Influence of CRHR1 Polymorphisms and Childhood Abuse on Suicide Attempts in Affective Disorders: A GxE Approach

**DOI:** 10.3389/fpsyt.2018.00165

**Published:** 2018-04-26

**Authors:** Birgit Ludwig, Klemens Kienesberger, Laura Carlberg, Patrick Swoboda, Alexandra Bernegger, Romina Koller, Qingzhong Wang, Michelle Inaner, Melanie Zotter, Nestor D. Kapusta, Helmuth Haslacher, Martin Aigner, Siegfried Kasper, Alexandra Schosser

**Affiliations:** ^1^Department of Psychiatry and Psychotherapy, Medical University of Vienna, Vienna, Austria; ^2^Department of Laboratory Medicine, Medical University of Vienna, Vienna, Austria; ^3^UAB Mood Disorder Program, Department of Psychiatry and Behavioral Neurobiology, University of Alabama at Birmingham, Birmingham, AL, United States; ^4^Department of Psychoanalysis and Psychotherapy, Medical University of Vienna, Vienna, Austria; ^5^Department of Psychiatry and Psychotherapy, Karl Landsteiner University for Health and Science, Tulln, Austria; ^6^Zentren für Seelische Gesundheit, BBRZ-Med, Vienna, Austria

**Keywords:** gene-environment interaction, childhood trauma, suicide attempt, affective disorders, CRHR1

## Abstract

**Background:** Previous studies have shown that the hypothalamus-pituitary-adrenal-axis (HPA-axis) is closely involved in the development of affective disorders. Given that early life events are also linked to dysregulation of the same system, there might be an association between childhood adversities and suicidal behavior in affective disorders, moderated by HPA-axis genes. We aimed to investigate a potential association between childhood trauma and previous suicide attempts in affective disorder patients, moderated by variants of the corticotropin-releasing hormone receptor 1 (CRHR1) gene.

**Methods:** The current pilot study is part of an ongoing study on suicidal behavior in affective disorders (VieSAD). Two hundred fifty eight Caucasian affective disorder patients were assessed at the Department of Psychiatry and Psychotherapy of the Medical University Vienna and the Karl Landsteiner University for Health and Science. An assemblage of psychiatric interviews was performed (e.g., SCAN, HAMD, SBQ-R, CTQ) and DNA samples of peripheral blood cells were genotyped with TaqMan® SNP Genotyping Assays (rs7209436, rs4792887, rs110402, rs242924, and rs242939).

**Results:** Neither genetic, nor haplotypic associations between CRHR1 polymorphisms and previous suicide attempts could be established for the present sample. Using a binary logistic regression model, significant gene-environment-interactions were found for the single nucleotide polymorphisms (SNPs) rs7209436 and rs110402, reflecting the impact of childhood trauma and CRHR1 polymorphisms on previous suicide attempts.

**Limitations:** A larger sample size will be required to ultimately elucidate the link between childhood trauma and the HPA axis in suicidal behavior.

**Conclusion:** This pilot study presents promising gene-environment-interaction findings in affective disorder patients with a history of suicide attempts.

## Introduction

Affective disorders represent a considerable burden to the global public health. By the year 2020, statistics of the WHO project Global Burden of Disease suggest that depression might be the second leading cause of disability worldwide (1).

Mental illness is present in almost 90% of all suicide cases, as shown by psychological autopsy studies (2, 3) and thereof affective disorders seem to have the strongest correlation with suicide (4).

Annually, an estimated 800,000 deaths are attributed to individuals taking their own life. Suicide was the second leading cause of death among 15–29-year-olds globally in 2015^1^. A European study shows that the suicide rate in Austria followed a significant downward trend in the past 30 years (5). Data gained from the beginning of the new century (2000–2010) shows that Austria's rates continued decreasing to an average of 26.1 per 100,000 persons for men, 8.2 for women, and 16.9 in the general population (6, 7).

Investigations of the inheritance of complex, quantitative traits such as affective disorders, family, twin and adoption studies show that the genetic contribution to affective disorders (“heritability”) ranges from 37% (8) in Major Depression Disorder (MDD) to 87% in Bipolar Disorder (BD) (9). Twin, adoption (10) and immigrant population studies (11) also suggest a genetic contribution to suicide. A population study including 11.4 million individuals found a twofold increased risk of suicide in children and a threefold risk in siblings of individuals who died by suicide, compared to children, and siblings of controls (12).

Recent genome-wide assessments of possible variations in patients with suicidal behavior indicate a genetic architecture of multiple genes with small effect sizes (13–15).

There are several hypotheses regarding the genetic substrate, respectively the risk-increasing alleles of suicidal behavior. Most findings relate to candidate genes of the serotonergic system (such as the serotonin transporters), genes involved in the hypothalamus-pituitary-adrenal-axis (HPA-axis) (CRHR1, CRHR2, FKBP5), neurotrophic genes such as BDNF (Brain-Derived-Neurotrophic-Factor) and noradrenergic genes like COMT (Catechol-O-Methyl-Transferase) (16).

It has been widely suggested that hyperactivity of the HPA-axis is implicated in the pathogenesis of both mood disorders (17, 18) and suicidal behavior (19–22). A reduction in negative feedback activation—experimentally achieved by long-term stress exposure—has been associated with depression-like rodent models (23). *In-vitro* studies also suggest that CRH signaling might be implicated in neurogenesis and memory formation (24). Finally, studies in clinical populations suggest that HPA-axis hyperactivity in adults is linked to childhood adversities and traumatic events (25).

One of the most promising genes in the context of suicidality is CRHR1. This gene encodes a G-protein coupled receptor that binds neuropeptides of the corticotropin releasing hormone family of the hypothalamic-pituitary-adrenal pathway^2^.

A Phase II clinical trial (NCT01018992) with a CRHR1-antagonist evaluating its efficacy in a sample of PTSD patients was recently completed; and while its overall-efficacy was not satisfactory, there was a significant treatment response in a subsample of patients who had experienced moderate to severe childhood abuse and were GG homozygotes for rs110402 (26). Hypothetically, CRHR1-antagonists—if consistently successful in trials, would be most effective in individuals with underlying alterations of the HPA-axis, reflecting a patient-tailored therapy model. Therefore, biomarkers would be needed identify patients responding to HPA-axis-treatment (27).

Direct or down-stream CRHR1 actions could be associated with suicidal states or suicidal behavior (28). Guillaume et al. (29) have shown that CRHR1 polymorphisms modulate the effect of childhood adversities on decision-making in suicide attempters (29). Another study from Sweden focused on the GxE-interactions between stressful live events and CRHR1 on suicide attempts and has shown sex-specific gene-environments associations in three Single Nucleotide Polymorphisms (SNPs) (30). Roy et al. (31) found CRHBP (high-affinity binding protein) variations to predispose, independently and additively, to suicidal behavior in individuals who had experienced childhood trauma (31). Recently, a strong association between CRHR1 polymorphism and suicide attempts in a Russian population was shown (32). Noteworthy, a study examining 235 HPA-axis single-nucleotide polymorphisms did not find associations, but suggested a trend between CRHR1 SNP (rs2664008), early childhood abuse and suicide attempts in bipolar patients (33).

Although affective disorders are the most common risk factor for suicide, suicidal behavior seems to be based on a distinct genetic makeup (34). Studies designed to specifically identify genes and polymorphisms of interest need to focus on patients with specific psychiatric disorders and compare them based on their suicidality.

Given the inconsistency of previous data the present study's aim is to investigate a possible association between childhood trauma moderated by Single Nucleotide-Polymorphisms (SNPs) of CRHR1 and lifetime suicide attempts in Caucasian patients with MDD or BD. Based on the literature mentioned above, we considered the gene CRHR1 to be the most promising candidate of the HPA-axis in this regard.

## Materials and methods

### Participants

A total of 258 subjects with affective disorders were collected at the Department of Psychiatry and Psychotherapy of the Medical University Vienna and the University Hospital Tulln, Lower Austria in the context of the Austrian Science Funds (FWF) funded study “VieSAD” (“Vienna Study on Genetics of Suicidal Behavior in Affective Disorders,” KLI°220). The investigation was carried out in accordance with the latest version of the Declaration of Helsinki and approval for the study was obtained from the Ethical Committee of the Medical University of Vienna (approval number EK 2013/2013) and the Ethical Committee of the federal state of Lower Austria (approval number GS4- EK-4/181/2012). The sample shown here constitutes only the first part of the ongoing VieSAD study and should be considered a pilot study. Caucasians aged from 18 to 65 years were included if they were diagnosed either BD or MDD as defined by ICD-10 and/or DSM-IV criteria. Exclusion criteria were psychotic symptoms or lifetime history of schizophrenia, primary substance abuse, pregnancy and breastfeeding. Diagnosis was affirmed by performing detailed clinical examination (SCAN – Schedules for Clinical Assessment in Neuropsychiatry – (35)) and suicidal behavior was assessed by VISURIAS (36), SBQ-R (Suicidal Behaviors Questionnaire-Revised) (37) and LPC – (Lifetime Parasuicide Count) (38). Two additional self-report scales were applied to screen (traumatic) life events, the CTQ-SF (short form of the Childhood Trauma Questionnaire, (39), and the BLEQ (Brief Life Events Questionnaire (40)). In order to screen for acute affective states, the HAMD (Hamilton Depression Scale (41)) and MADRS (Montgomery-Asberg Depression Scale (42)), as well as the YMRS (Young Mania Rating Scale (43)) were applied when blood for genotyping was drawn. Comorbidities were monitored, as well as weight, height, and BMI (Body Mass Index). In a face-to-face interview, patients were informed about the study and signed a written consent form. Interrater reliability was guaranteed by extensive interview training and videotaping. All interviewers followed Good Clinical Practice criteria.

### Statistical analyses

Statistical analyses were conducted using SPSS 22.0 (IBM, Armonk USA). Continuous data were presented as mean and standard deviation, respectively with confidence intervals. Categorical data were given as counts and percentages. Fisher's exact test and χ^2^ analysis were calculated to test the equal distribution of categorical variables. Differences between groups were assessed by means of One-way ANOVA testing for variables following a normal distribution, or Mann-Whitney *U*-Test for skewed data. Normal distribution of the variables was tested by Kolmogorov-Smirnov test. All test results were interpreted two tailed with a significance level established at *p* < 0.05. Bonferroni correction was used for multiple testing correction.

For comparisons of genotype frequencies within the groups of suicidal phenotypes and gender as categorical variables, we performed χ^2^ tests as well as Fisher's exact test. To test for Hardy–Weinberg equilibrium (HWE), online software provided by the Helmholtz Center Munich (https://ihg.gsf.de/cgi-bin/hw/hwa1.pl) was used. Linkage disequilibrium (LD) analysis was performed using Haploview (44): five SNPs of the CRHR1 gene were analyzed; frequencies lower than 1% were excluded from the analysis. Power analyses were performed using G^*^Power 3.1.9.2; effect sizes (w) and power (1-ß error probability) were determined for each individual SNP, assuming a sample size of 250, degree of freedom of 2 and α-error probability of 0.05 (45). Effect sizes and power of the individual SNPs are presented in Table 3. While we found effect sizes between 0.05 and 0.14 for the SNPs investigated in the current study, we have 0.82 power to detect effect sizes of 0.2 and 0.72 power to detect effect sizes of 0.18 in our sample of 250 individuals.

Similar to previous studies on suicide (31, 33, 46) we used a logistic regression-based approach to further explore the relationship between suicide and environmental factors. GxE interactions between the variables CRHR1 polymorphisms and CTQ score were evaluated in PLINK (http://zzz.bwh.harvard.edu/plink/) (47). Assuming additive genetic model, previous suicide attempts were established as the dependent variable and the total CTQ-score and genotypes (all SNPs in separate models) as independent variables. The variable previous suicide attempt was operationalized by integrating various items to one (SBQ 01, SCAN 6.011, VISURIAS A1). Thus, gathering additional information to the circumstances and reasoning behind the suicide attempt results in a more precise and homogeneous variable. We also controlled for sex as a covariate.

## Genotyping

Genomic DNA was isolated from whole blood samples using E.Z.N.A. Blood DNA Mini Kit (Omega bio-tek) according to the manufacturer's protocol. Five SNPs (rs7209436, rs4792887, rs110402, rs242924, and rs242939) of the CRHR1 gene were chosen in accordance with previous literature findings (26, 30, 48). CRHR1 is located on chromosome 17 and the SNPs in the Genome Reference Consortium Human Build 38 are positioned as follows: rs7209436 (C/T): 45792776 on GRCh38 (intron); rs4792887 (C/T): 45799654 on GRCh38 (intron); rs110402 (C/G/T): 45802681 on GRCh38 (intron); rs242924 (G/T): 45808001 on GRCh38 (intron); rs242939 (C/T): 45818213 on GRCh38 (intron). These corresponding SNPs of the CRHR1 gene were genotyped using a Custom TaqMan®-Genotyping Assay (Applied Biosystems, Rotkreuz, Switzerland) containing specific primers and fluorescence labeled probes for the analysis. These assays are commercially available from Applied Biosystems (Assay IDs: rs7209436: C___1570087_10, rs4792887: C__27895630_10, rs110402: C___2544843_10, rs242924: C___2257689_10, and rs242939: C___2544833_10). PCR was performed in a 384-well format according to a protocol provided by the manufacturer. Shortly, in a total volume of 10 μL containing 5 μL TaqMan Universal PCR Mastermix (2×), No AmpErase UNG (Applied Biosystems), 0.5 μL working stock (20×) of Custom TaqMan®-Genotyping Assay (Applied Biosystems) and 2.25 ng diluted patients' DNA were added. PCR runs were performed on an Applied Biosystems 7900HT Fast Real-Time PCR System (Applied Biosystems) using the following conditions: enzyme activation at 95°C for 10 min, followed by 40 cycles of 92°C for 15 s and 60°C for 1 min. Allelic discrimination by qualitative detection of fluorescence labeled probes was accomplished by Applied Biosystems sds 2.3 software (Applied Biosystems).

## Results

In total, 258 patients were recruited, 3 patients had to be excluded from further analyses because of incomplete interview data, resulting in a total of 255 patients. The mean age of the participants was 46.5 years (*SD* = ±14.3). Descriptive statistics of the sample are presented in Table 1. No significant demographic risk factors for suicide attempt could be identified in the present sample: unemployment showed a tendency but lost significance after correction for multiple testing. These findings are published in a previous report (49). Childhood maltreatment assessed by the CTQ is presented in Table 2, showing tendencies of higher scores for females in comparison to males in all subscales, reaching statistical significance for sexual abuse only, which has been thoroughly discussed in the previous report (49).

**Table 1:** Descriptive statistics of diagnosis and suicidal behavior (suicide attempt and non-suicidal self-injury).

	**Female (*n* = 145)**		**Male (*n* = 110)**		**Whole Sample (*n* = 255)**	
MDD	*n* = 121	83.4%	*n* = 90	80.2%	*n* = 211	81.4%
BD	*n* = 24	16.5%	*n* = 20	18.9%	*n* = 44	17.1%
Suicide attempt	*n* = 45	31.0%	*n* = 25	22.7%	*n* = 70	27.5%
Non-suicidal self-injury	*n* = 34	23.4%	*n* = 13	11.8%	*n* = 47	18.4%

**Table 2:** Social demographic and clinical characteristics of the patient sample.

	**Whole sample (*n* = 255)**	**Male (*n* = 110)**	**Female (*n* = 145)**	***p***	***Z***
CTQ total score *M* ± *SD*	52.42 ± 20.14	49.49 ± 18.37	55.84 ± 21.61	0.06	−0.19
CTQ emotional abuse	9.97 ± 5.16	9.30 ± 4.66	10.49 ± 5.42	0.115	−1.58
CTQ physical abuse	7.32 ± 4.20	7.08 ± 3.69	7.50 ± 4.58	0.881	−0.15
CTQ sexual abuse	7.08 ± 5.04	5.85 ± 3.32	8.02 ± 5.89	**<0.001**	−3.60
CTQ emotional neglect	12.41 ± 5.79	11.91 ± 6.12	12.80 ± 5.52	0.138	−1.48
CTQ physical neglect	7.60 ± 3.48	7.48 ± 3.43	7.70 ± 3.53	0.654	−0.45

*CTQ, childhood trauma questionnaire. Bold indicates a significant (>0.05) p-value*.

**Figure 1:**
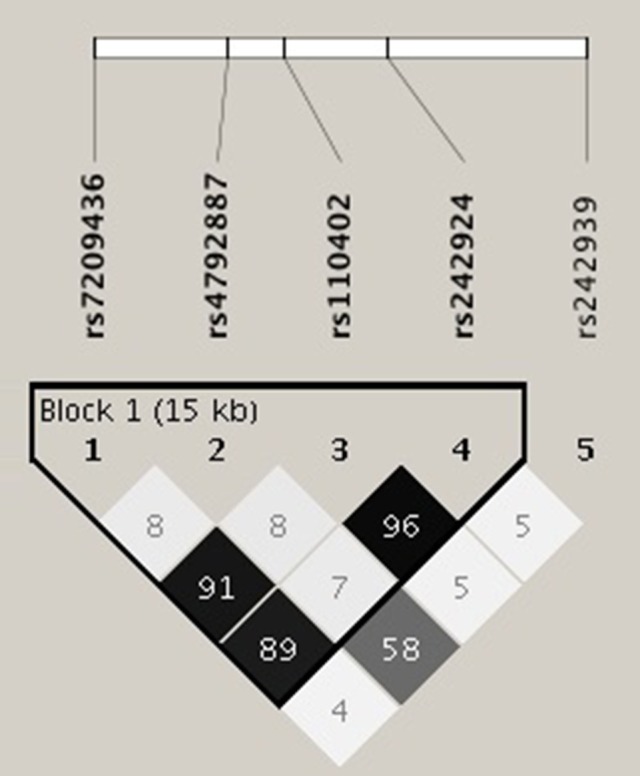
Linkage disequilibrium (LD) analysis was performed using Haploview software. The selection criteria for haplotypes used in the analyses were adjacent SNPs with pairwise *r*^2^ > 0.80; also only haplotypes with frequencies above 0.03 were tested. According to the selection criteria, two of five SNPs of the CRHR1 gene (rs110402 and rs242924) with strong *r*^2^ > 0.80 were in one block.

The primary goal was to test for associations of CRHR1 SNPs with (history of) suicide attempt in a cohort of patients suffering from affective disorders (MDD and BD). A total of 27.5% of the patients had a personal history of suicide attempt(s) and 18.4% had a history of non-suicidal self-injury (Table 1). HWE was tested both for patients with and without previous suicide attempt(s) applying Fisher's exact test (https://ihg.gsf.de/cgi-bin/hw/hwa1.pl) and no deviation was noted for the PWSA whereas rs4792887 and rs242939 of the PSA were significant for HWE deviation (Table 3). Lifetime history of suicide attempt (yes/no) was analyzed as a dichotomous trait applying the standard chi-square statistics (and Fisher's exact test for the SNPs with allele frequency <5) finding neither genotypic nor allelic association with any of the tested SNPs (see Table 3). The selection criteria for haplotypes used in the haplotype analyses were adjacent SNPs with pairwise *r*^2^ > 0.80. In the analysis, haplotypes with frequencies above 0.03 were tested. According to the selection criteria, two SNPs (rs110402 and rs242924) with strong *r*^2^ > 0.80 were in one block. (Figure 1) Further association analysis for the adjacent block did not reveal any significant associations between the two most frequent haplotypes A-T/G-G and lifetime history of suicide (χ^2^ = 0.62, *p* = 0.43) (Table 4).

**Table 3:** Personal history of Suicide Attempt vs. no history of suicide attempt, single marker analyses were established with standard chi-squared testing.

**CRHR1**			**Power analysis**		**Genotypes**		**Alleles**		**HWE**	
**SNP ID**	**N° of patients with suicide attempts**	**N° of patients without suicide attempts**	**Effect size (w)**	**Power (1–ß-error)**	**χ^2^**	***p***	**χ^2^**	***p***	**Patients with suicide attempts (*p*-value)**	**Patients without suicide attempts (*p*-value)**
rs7209436	69 (CC: 26, TC: 29, TT: 14)	181 (CC: 60, TC: 85, TT: 36)	0.048	0.10	0.57	0.75	0.16	0.69	0.27	0.55
rs4792887	70 (CC: 58, CT: 9, TT: 3)	181 (CC: 146, CT: 34, TT: 1)	0.140	0.49	4.89^*^	0.07^*^	0.06	0.80	0.024^*^	1.00^*^
rs110402	70 (GG: 26, AG: 29, AA: 15)	181 (GG: 56, AG: 83, AA: 42)	0.060	0.12	0.89	0.64	0.59	0.44	0.21	0.30
rs242924	70 (GG: 24, TG: 32, TT: 14)	181 (GG: 55, TG: 83, TT: 43)	0.047	0.10	0.56	0.76	0.55	0.46	0.58	0.29
rs242939	70 (TT: 62, CT: 6, CC: 2)	181 (TT: 154, CT: 26, CC: 1)	0.119	0.37	3.51^*^	0.12	0.05	0.83	0.03^*^	1.00^*^

CRHR1, Corticotropin Releasing Hormone Receptor 1; SNP, Single Nucleotide Polymorphism; N°, Number; HWE, Hardy–Weinberg-Equilibrium.

* *calculated with Fisher's Exact Test. Bold indicates a significant (>0.05) p-value*.

**Table 4:** Estimated frequency of haplotypes and association significance: Chi-Square values and Pearson's *p*-values (global and individual) and Odds Ratio with 95% Confidence Interval.

**No. of Markers**	**Haplotype**	**Global *p***	**Alleles increasing in cases**	**Estimated haplotype frequency %**		**χ2**	***p***	**Odds Ratio [95%CI]**
				**Patients with previous suicide attempts**	**Patients without previous suicide attempts**			
2	rs110402–rs242924	0.43	A–T	0.41	0.46	0.62	0.43	0.851 [0.569–1.273]
2	rs110402–rs242924	0.43	G–G	0.57	0.54	0.62	0.43	1.175 [0.785–1.756]
2	rs110402–rs242924	–	G–T	0.02	0.006	–	–	–
2	rs110402–rs242924	–	A–G	0.007	0.000	–	–	–

A binary logistic regression model was established including suicide attempt as the dependent variable and SNP genotypes and childhood trauma scores as covariates (Table 5). A significant GxE interaction could be established for three SNPs rs7209436 (*p* = 0.009, *Z*-value = 2.61), rs110402 (*p* = 0.009, *Z*-value = 2.61), and rs242924 (*p* = 0.01, *Z*-value = 2.55); rs7209436 and rs110402 remained significant after Bonferroni correction for multiple testing (<0.01).

**Table 5:** Logistic regression model for suicide attempt with SNP-genotype and CTQ-score as predictor variables.

**SNP**	**Patient without previous suicide attempts (181)**		**Patients with previous suicide attempts (70)**		**GxE interaction**	
	**Coef**	**SE**	**Coef**	**SE**	***Z*-value**	***p*-value**
rs7209436	0.154	0.19	1.575	0.64	2.61	**0.009**
rs4792887	-0.285	0.34	1.24	0.95	-1.51	0.13
rs110402	0.27	0.19	-1.43	0.63	2.61	**0.009**
rs242924	0.266	0.188	-1.454	0.65	2.55	**0.01**
rs242939	-0.221	0.37	0.87	1.14	-0.912	0.36

*SE, standard error; Coef, beta coefficient. Bold indicates a significant (>0.05) p-value*.

## Discussion

In this study, we examined a possible influence of childhood trauma and CRHR1 polymorphisms on lifetime suicidal behavior in a sample of patients with affective disorders.

One of the main hypotheses of the current study was to explore a genetic association of CRHR1 and suicide attempts. Several studies linked suicide attempts to polymorphisms of the CRHR1 gene as shown in previous reviews (28, 32). However, in this pilot study these findings could not be replicated, likely limited by the current sample size and the narrow selection of SNPs.

In addition to the SNP genotypes, we investigated whether there was a significant contribution of the most frequent haplotypes to lifetime suicide risk. Linkage disequilibirium analysis showed one block of non-random association of alleles between rs110402 and rs242924; haplotype analysis of this block revealed no significant associations between AT/GG and lifetime suicide attempts. Given the fact that this is an ongoing study, additional SNPs and haplotypes will be analyzed in the near future.

Using a binary logistic regression model to test for a gene-environment-interaction we found a significant interaction of childhood trauma and CRHR1 polymorphisms on the history of suicide attempts. Both rs7209436 and rs110402 reached significance and remained significant after Bonferroni correction for multiple testing. A very similar study—regarding the coefficient (CTQ score) and the dependent variable (suicide attempt)—published by Roy et al. (31), investigated GxE interactions for Corticotropin Releasing Hormone Binding Protein (CRHBP), CRH, CRHR,1 and CRHR2 in a sample of African American patients. Interestingly, they found the African-American specific CRHR1 SNP rs9900679 to be a significant protective factor for suicide attempt, but no significant associations were found for the CRHR1 SNP rs110402 (31). The second finding stands in contradiction to ours and might be explained by the different ethnicities that were investigated. Recently, Ben-Efraim et al. (30) found sex-specific gene-environment interactions for several SNPs, including rs7209436. We were able to replicate this association between rs7209436 and suicide attempts independently from sex. However, limiting comparisons, the study designs vary considerably regarding the targeted population and also regarding the operationalization of the environment variable (30). A recently published candidate gene association study, with a larger sample of BD patients only, tested 19 HPA-axis genes for interaction with early childhood abuse. No significant results, only a trend of the CRHR1-SNP rs2664008 was found comparing suicide attempters to non-attempters among 1288 bipolar patients (33). Interestingly, a very recent clinical trial for a CRHR1-antagonist only proved efficacy in the subsample of patients who showed one of the polymorphisms that we identified in our study (rs110402) and who reported abuse in their childhood (26).

The sample size limits to reveal small genetic effects as expected to be involved in suicidal behavior, although this limitation might be counteracted by detailed phenotypic classification as performed in the current study (50). Besides that, only a selection of SNPs of the CRHR1 gene was analyzed within this pilot study.

Candidate gene association studies have been conducted since the 1980s and helped studying complex diseases for which many potential candidate genes exist. One of the well-known limitations of these study designs is the lack of successful replication studies. Thus, replication of the current findings is mandatory to draw distinct conclusions. Another limitation of this study is the retrospective assessment of suicidality; all gathered data is based on interviews, therefore we cannot guarantee for the accuracy of reported suicide attempts. We attempted to circumvent this limitation by integrating various interview scales and eliminating patient data when incongruences surfaced. Still, self-report instruments convey a certain potential of inaccuracy. Although a previous suicide attempt is the best predictor for a future attempt (51), no ultimate predictive factors have been identified yet. In this respect the operationalization of suicide in this study only represents an approximation to the concept.

A clear strength of this study is the specific phenotypic definition of our sample (only affective disorder patients, detailed description of suicidal behavior), which results in increased homogeneity of the sample. This study design allows us to precisely describe the suicidal phenotype and prevent interference of the confounding affective disorders phenotype.

A larger sample size, broader SNP selection and replication of the current findings are necessary to ultimately elucidate the link between childhood trauma and the HPA axis in suicidal behavior with more certainty.

## Author contributions

BL conception and design, acquisition of data, analysis and interpretation of data, manuscript writing, final approval of the version to be published; KK acquisition of data, analysis and interpretation of data, manuscript writing, final approval of the version to be published; AB acquisition of data, manuscript revision, final approval of the version to be published; LC acquisition of data, analysis and interpretation of data, manuscript revision, final approval of the version to be published; PS acquisition of data, manuscript revision, final approval of the version to be published; RK acquisition of data, manuscript revision, final approval of the version to be published; MI and MZ acquisition of data, manuscript revision, final approval of the version to be published; QW interpretation of data, manuscript revision, final approval of the version to be published; NK, MA, and SK conception and design, acquisition of data, analysis and interpretation of data, manuscript revision, final approval of the version to be published; HH analysis and interpretation of data, manuscript writing, final approval of the version to be published; AS principal investigator of the VieSAD study, conception and design, acquisition of data, analysis and interpretation of data, manuscript revision, final approval of the version to be published. All authors agree to be held accountable for all aspects of the manuscript.

### Conflict of interest statement

SK has received grant/research support from Bristol Myers-Squibb, Eli Lilly, GlaxoSmithKline, Lundbeck, Pfizer, and Servier; he has served as a consultant or on advisory boards for AstraZeneca, Bristol-Myers Squibb, Eli Lilly, GlaxoSmithKline, Janssen, Lundbeck, Novartis, Pfizer, Schwabe and Servier; and he has served on speakers' bureaus for Angelini, AOP Orphan Pharmaceuticals AG, AstraZeneca, Bristol Myers-Squibb, Eli Lilly, Janssen, Lundbeck, Neuraxpharm, Pfizer, Pierre Fabre, Schwabe, Servier. The other authors declare that the research was conducted in the absence of any commercial or financial relationships that could be construed as a potential conflict of interest.

## Footnotes

^1^[Dataset] WHO. *Suicide. Fact sheet*. World Health Organization.

^2^[Dataset] NCBI-Gene. *CRHR1 corticotropin releasing hormone receptor 1 [ Homo sapiens (human) ] Gene ID: 1394*.

## Abbreviations

BD: Bipolar Disorder
CRHR1: Corticotropin releasing hormone receptor 1
CTQ: Childhood Trauma Questionnaire
GxE: Gene-Environment
HPA axis: hypothalamic–pituitary–adrenal axis
MDD: Major Depressive Disorder
SNP: Single Nucleotide Polymorphism.
